# How Phagocytes Acquired the Capability of Hunting and Removing Pathogens From a Human Body: Lessons Learned From Chemotaxis and Phagocytosis of *Dictyostelium discoideum* (Review)

**DOI:** 10.3389/fcell.2021.724940

**Published:** 2021-08-20

**Authors:** Xuehua Xu, Miao Pan, Tian Jin

**Affiliations:** Chemotaxis Signal Section, Laboratory of Immunogenetics, NIAID, NIH, Rockville, MD, United States

**Keywords:** chemotaxis, phagocytosis, innate immunity, *D. discoideum*, phagocyte

## Abstract

How phagocytes find invading microorganisms and eliminate pathogenic ones from human bodies is a fundamental question in the study of infectious diseases. About 2.5 billion years ago, eukaryotic unicellular organisms–protozoans–appeared and started to interact with various bacteria. Less than 1 billion years ago, multicellular animals–metazoans–appeared and acquired the ability to distinguish self from non-self and to remove harmful organisms from their bodies. Since then, animals have developed innate immunity in which specialized white-blood cells phagocytes- patrol the body to kill pathogenic bacteria. The social amoebae *Dictyostelium discoideum* are prototypical phagocytes that chase various bacteria via chemotaxis and consume them as food via phagocytosis. Studies of this genetically amendable organism have revealed evolutionarily conserved mechanisms underlying chemotaxis and phagocytosis and shed light on studies of phagocytes in mammals. In this review, we briefly summarize important studies that contribute to our current understanding of how phagocytes effectively find and kill pathogens via chemotaxis and phagocytosis.

## Introduction

How eukaryotic cells interact with bacteria and viruses is a fundamental question in biology. Eukaryotic cells and their interactions with other life forms began when single-cell organisms such as protozoa appeared about 2.5 billion years ago. Since then, multicellular organisms with increasingly complex genomes have developed innate immune systems in which specialized phagocytic cells, such as neutrophils and macrophages in humans, patrol the body to detect, recognize and eliminate invading pathogens. Specialized eukaryotic cells that can recognize foreign objects by migrating toward them and engulfing them were discovered by Elie Metchnikoff in 1882 (Beck and Habicht, 1996). One day, he pierced the larva of a starfish with a rose thorn. When he examined the transparent larva the next day, he saw many cells covering the thorn and trying to engulf it. He imagined that these cells migrated from afar toward foreign invaders, a cellular process known as chemotaxis, and attempted to engulf the invaders, a cellular process known as phagocytosis. Both processes were also described in some specialized cells by others at the time. Engelmann T. W. (1881) and Pfeffer W. F. (1884) described chemotaxis of bacteria (Mc, 1946; Drews, 2005), and Leber T. (1888) discovered that leukocytes migrated in straight paths to sites of rabbit corneal irritation (Mc, 1946). Those observations gave rise to the hypothesis that diffusible substances guide the migration (chemotaxis) of those cells. Many reports of phagocytosis described the uptake of microorganisms, red cells, or various particles by cells in different animal systems. For example, Hayem (1870) and Klebs (1872) observed bacteria in white blood cells; Waldeyer (1876) found bacteria in peritoneal pus cells, Koch (1876) found anthrax bacilli in spleen and lymph node cells, and Osler (1875) reported that human alveolar cells engulfed coal dust particles (Ambrose, 2006). Metchnikoff proposed that recognizing foreign substances and engulfing them represents a fundamental mechanism by which organisms in the animal kingdom defend themselves against infections. Therefore, he created the discipline of cellular immunology, which is now called innate immunity.

It was generally believed that human phagocytes use at least two different types of receptors for defense against bacteria pathogens: one for detecting and chasing pathogens via chemotaxis and another for recognizing and engulfing them via phagocytosis. Detection and chasing are facilitated by chemoattractant G-protein-coupled-receptors (GPCRs) (Jin et al., 2008; Metzemaekers et al., 2020), whereas recognition and engulfing employ pattern-recognition receptors (PRRs), such as Toll-like receptors (TLRs), scavenger receptors, and C-type lectin receptors, as well as phagocytic receptors that include complement receptors and Fcγ receptors (Flannagan et al., 2012; Amarante-Mendes et al., 2018).

The social amoebae *Dictyostelium discoideum* inhabit soil and feed on diverse bacterial species, including gram-positive and gram-negative bacteria (Vogel et al., 1980; Gerisch, 1982; Devreotes and Zigmond, 1988; Soldati and Cardenal-Munoz, 2019). The amoebae locate bacteria by detecting metabolites, such as folic acid; move toward the bacteria via chemotaxis; then engulf the bacteria by recognizing their surface molecules and consume them through phagocytosis. Over the years, many molecular components involved in eukaryotic chemotaxis and phagocytosis have been discovered. Studies in *D. discoideum* have helped us to uncover molecular components and pathways and to develop new techniques. Importantly, molecular mechanisms are evolutionarily conserved allowing us to apply knowledge gained from the model organism to the study of chemotaxis and phagocytosis in human phagocytes. In this review, we will summarize landmark discoveries in phagocytic cells that shape our knowledge of innate immunity and highlight important studies in *D. discoideum* that provide novel insights to our current understanding of chemotaxis and phagocytosis.

## Chemoattractants and Chemokines

The idea that eukaryotic cells can migrate in a gradient of diffusible substances and move toward their sources took root before 1900 (Mc, 1946). In 1882, Metchnikoff first noted that cells migrated from afar and covered a rose thorn inserted into the larva of a starfish (Beck and Habicht, 1996), and Leber (1888) discovered that leukocytes migrated in straight paths to sites of rabbit corneal irritation (Mc, 1946). Those observations gave rise to the hypothesis that diffusible substances generated by a foreign object or at the site of injury establish chemical gradients that guide cell migration.

It had been known for many years that some motile cells, including mammalian leukocytes and metazoan cells, move toward the sources of diffusible chemicals, but the chemical nature of the substances was not known and could be not be properly analyzed because experimental techniques did not yet exist. In 1962, Boyden reported a technique, now known as the Boyden chamber assay, to test for chemotactic activity of diffusible substances (Boyden, 1962). The first chemoattractant to be reported for eukaryotic cells was 3′5′-cyclic adenosine monophosphate (cAMP) for *Dictyostelium discoideum* (Table 1). In 1947, Bonner reported that the eukaryotic social amoebae, *D. discoideum*, migrate robustly toward the source of diffusible chemicals (Bonner, 1947). In 1969, it was discovered that cAMP is a chemoattractant for the social amoeba (Konijn et al., 1969). A couple of years later, the Bonner group found that the folic acid released from bacteria serves as another chemotactic substance used by *D. discoideum* cells to hunt their bacterial food (Pan et al., 1972). A chemotactic factor for leukocytes was found in the complement system of serum in 1968, later known as C5a (Snyderman et al., 1968). In 1975, N-formyl peptides, which are released from bacteria, were identified as chemoattractants for leukocytes (Schiffmann et al., 1975). By the early 1980s, the “classical” chemoattractants, including N-formyl peptides, C5a, and the lipid mediator leukotriene B4 (LTB4) (Borgeat et al., 1976; Fernandez et al., 1978), had been identified, and the cytokine-like-chemoattractants, chemokines, would be discovered in the coming years.

**TABLE 1:** Chemoattractants, chemokines, and their receptors are listed in the table.

Chemoattractants	Receptors	Organisms
cAMP	cAR1-cAR4	*D. discoideum*
Folate	fARl	*D. discoideum*
C5a	C5aR	Human
N-formyl peptides (fMLP)	FPRl and FPR2	Human
LTB4	BLTl and BLT2	Human
CXCL8 (IL-8)	CXCRl and CXCR2	Human
CCL2 (MCP-1)	CCR2	Human
CXCL10 (IP-10)	CXCR3A and CXCR3B	Human
CCL3L1 (LD78)	CCRl, CCR3, and CCRS	Human
CCL1 (I-309)	CCR8	Human

The chemokine field started with the cloning of the human gene encoding IL-8, also known as CXCL8 (Baggiolini, 2015). Several groups independently cloned the human gene encoding CXCL8 (Walz et al., 1987; Yoshimura et al., 1987). The amino acid sequences of CXCL8 and monocyte chemoattractant protein-1 (MCP-1, also known as CCL2) contained either CXC or CC, which led to the identification of the protein family of chemokines with at least two CXC or CC subgroups (Matsushima et al., 1989; Yoshimura et al., 1989). Interestingly, chemokine-like proteins, such as IP-10 (CXCL10) (Luster et al., 1985), LD78 (CCL3L1) (Obaru et al., 1986), and the mouse ortholog of human I-309 (CCL1) (Miller et al., 1989), had been identified several years before CXCL8. However, their chemoattractant activity remained unclear until the discovery of CXCL8.

By the early 1990s, the new field of chemokines took off. Today, we can easily identify genes encoding typical chemokines in genome sequences due to their size (about 70–90 amino acids) and conserved N-terminal, cysteine motifs (Murphy et al., 2000). Based on the number and spacing of cysteines in these motifs, chemokines have been classified into C, CC, CXC, and CX3C subfamilies. There are more than fifty chemokines that are produced by leukocytes and tissue cells. Some chemokines are involved in normal housekeeping functions, such as the maturation of leukocytes in the bone marrow, the trafficking and homing of lymphocytes, and the regeneration of circulating leukocytes, while others orchestrate inflammatory responses for the recruitment of immune cells to the inflamed regions. Interestingly, some chemokines have been shown to also play significant roles in cancer metastasis (Jin et al., 2008; Li et al., 2013).

## Chemoattractant- and Chemokine-Receptors

The molecular identities of chemoattractant receptors for eukaryotic cells began to be revealed in the late 1980s when DNA cloning techniques became widely available for laboratories. The first breakthrough was the discovery of a cAMP chemoattractant receptor cAR1 in *D. discoideum* and the realization that it belongs to the family of G protein-coupled receptors (GPCRs) (Table 1), which contains seven transmembrane domains and (Klein et al., 1988). A couple of years later, the receptors for classical chemoattractants, such as fMLP and C5a, had been cloned and were found to be GPCRs (Boulay et al., 1990; Gerard and Gerard, 1991). Before the identification of the IL-8 (CXCL8) receptor, evidence suggested that the putative receptor would be a GPCR since activation of neutrophils by IL-8 (CXCL8) was shown to be inhibited by pertussis toxin, a drug blocking GPCR/Gi signaling. However, single transmembrane tyrosine kinase receptors, such as the receptor for platelet-derived growth factor (Williams, 1989), can mediate chemotaxis, therefore it was speculated that tyrosine kinase receptors could also detect other protein-ligands, such as chemokines, at the time.

In 1991, two back-to-back papers reported the discoveries of two GPCRs as IL-8 receptors (Holmes et al., 1991; Murphy and Tiffany, 1991). The two receptors are similar but not identical, sharing 77% amino acid identity. Each of them triggered transient Ca^2+^ responses upon IL-8 stimulation and had a high affinity for IL-8 (Kd = 2 nM). They were named IL-8RA and IL-8RB at the time and are now known as CXCR1 and CXCR2. Currently, about twenty chemokine GPCRs mediate signal events induced by more than 50 chemokines (Murphy et al., 2000). Interestingly, chemokines and their receptors, unlike other types of GPCRs, have overlapping specificities for each other, and many chemokine-receptor-mediated signals appear to be redundant. It became a paradigm that phagocytes use GPCRs to detect diffusible chemicals (both classical chemoattractants and chemokines) and to mediate signaling pathways that control the reorganization of the actin cytoskeleton for cell migration toward the sites of infection and pathogens via chemotaxis. Once reaching the pathogens, these phagocytes employ pattern-recognition receptors (PRRs) to recognize surface molecules of the pathogens and to mediate signaling pathways that regulate the reorganization of the actin cytoskeleton for the engulfment of particles via phagocytosis (Greenberg, 1995; Flannagan et al., 2012; Kaufmann and Dorhoi, 2016).

## Phagocytosis and Opsonic- and Non-Opsonic Receptors

Phagocytosis was observed by Oster in 1875 and others, and later was studied and named by Metchnikoff in 1883 (Ambrose, 2006). Phagocytosis is a process of sensing and engulfing particles larger than 0.5 μm. The particle is internalized into an organelle, called a phagosome. The phagosome fuses with lysosomes to become a phagolysosome, which contains enzymes that can degrade the particle (Flannagan et al., 2012; Kaufmann and Dorhoi, 2016). Phagocytosis is a basic process for obtaining nutrition in unicellular organisms, such as free-living amoebae of *D. discoideum* (Dunn et al., 2017; King and Kay, 2019), and for the elimination of foreign microorganisms in multicellular organisms by a group of cells, such as neutrophils and macrophages in mammals.

Detection of the target particles is the first step of phagocytosis. In 1903, Wright and Douglas discovered that the uptake of staphylococci by human leukocytes is enhanced by serum (Wright et al., 1989). They concluded that blood fluids have an opsonic power that modifies bacteria to be ingested by the leukocytes. The idea that phagocytosis begins with pathogenic bacteria being labeled with opsonins–host-derived proteins that bind specific receptors on phagocytes–was widely accepted. Nowadays it is well known that the major opsonins promoting efficient phagocytosis by leukocytes are complement components and immunoglobulin G (IgG) antibodies (Uribe-Querol and Rosales, 2020). These opsonins and their receptors are the best studied.

Complement is a system of more than 30 proteins in the plasma and on the cell surface, and it constitutes more than 15 of the globular fractions of plasma (Dunkelberger and Song, 2010). The complement system comprises vital components of innate immunity and influences T- and B-cell biology and adaptive responses. A major role of complement in innate immunity is providing complement opsonins, such as C4b, C3b, and C3bi, to decorate the surface of microorganisms, and phagocytes recognize these marked microorganisms via the complement receptors (CRs), including CR1, CR2, CR3, and CR4, and then eliminate them via phagocytosis (Walport, 2001a,b; Dunkelberger and Song, 2010). IgG antibodies are another family of opsonins that bind to the surface of pathogenic microorganisms, and on human cells, IgG receptors are Fcγ receptors, such as FcγRI, FcγRII, and FcγRIII (Uribe-Querol and Rosales, 2020). Fcγ receptors specifically bind to the Fc part of IgG molecules to form clusters on the surface of phagocytes, and these clusters then trigger phagocytosis and other cellular responses.

Janeway Jr. (1989) proposed that innate immune cells must express receptors that recognize specific molecules on foreign microorganisms. Since then, receptors directly recognizing pathogen-associated-molecular-patterns (PAMPs) have been discovered, and these so-called pattern recognition receptors (PRRs) are proteins capable of binding to molecules associated with pathogens (Walsh et al., 2013; Amarante-Mendes et al., 2018). PRRs are found associated with different subcellular compartments, such as on cellular and endosomal membranes, in the cytosol, and the bloodstream as the secreted forms. Four main classes of PRRs are recognized, including the Toll-like receptors (TLRs), the nucleotide-binding oligomerization domain (NOD)-leucine-rich-repeats (LRRs)-containing receptors (NLRs), the retinoic acid-inducible gene 1 (RIG-1)-like receptors (RLRs), and the C-type lectin receptors (CLRs). Some of them, such as TLRs, can bind to PAMPs but cannot induce phagocytosis, while others, such as some CLRs (Dectin-1, Dectin-3, and DC-SIGN) can induce phagocytosis; the latter type is called non-opsonic receptors.

When a particle is recognized by either opsonic- or non-opsonic receptors, many signaling pathways in phagocytic cells are activated, resulting in reorganization of the actin cytoskeleton and the formation of a phagocytic cup around the particle for engulfment. Opsonic- and non-opsonic receptors, unlike chemoattractant GPCRs that have seven transmembrane domains, are single transmembrane proteins with extracellular ligand-binding domains and intracellular signaling domains (Greenberg, 1995; Greenberg and Grinstein, 2002; Flannagan et al., 2012). Activation of these receptors mediates phosphorylation of tyrosine residues on the intracellular domain that induces signaling events that lead to the rearrangement of the actin cytoskeleton for the engulfment of particles. It appeared that chemotaxis and phagocytosis are two distinct cellular processes that are sequentially connected by phagocytes during the fight against pathogens. Studies of *D. discoideum* suggest that chemotaxis and phagocytosis may be originated from one cellular process in ancient phagocytes.

## Chemotaxis of *D. discoideum* and Leukocytes

Before 1900, it was known that leukocytes migrated in straight paths to inflamed sites of rabbit corneal irritation (Mc, 1946). Leukocytes are amoeboid cells, and their movement closely resembles that of the amoebae of *D. discoideum* (Devreotes and Zigmond, 1988). In the 1970s, Gerisch used time-lapse microscopy to directly observe the behaviors of *D. discoideum* cells in response to cAMP stimuli (Gerisch et al., 1975). In response to a uniform cAMP increase, the cells became elongated and polarized, with clear leading fronts and trailing ends, and their random motility increased, a behavior termed chemokinesis. When placed in a cAMP gradient, the cells instantly migrated toward the source of cAMP by extending pseudopods (leading fronts) followed by retraction of the trailing ends (Gerisch et al., 1975; Gerisch, 1982; Swanson and Taylor, 1982). Zigmond observed movements of individual leukocytes in response to chemical gradients using time-lapse microscopy (Zigmond, 1974, 1978). Together, the studies of *D. discoideum* and leukocytes showed that these amoeboid cells can initiate locomotion by extending pseudopods in the direction of a higher concentration of chemoattractant (Devreotes and Zigmond, 1988).

Based on the observations, two different models have been proposed to explain how cells measure gradients (Parent and Devreotes, 1999; Rappel et al., 2002). In the first model, a cell detects changes in chemical concentration over time only and adjusts its movement in the chemical gradient based on the change in concentration it detects as it moves (Berg, 1975). Bacteria employ this temporal mechanism of chemotaxis (Berg, 1975). In the second model, a cell simultaneously compares differences in chemoattractant concentration around its perimeter, detecting spatial changes (Parent and Devreotes, 1999). Using this spatial mechanism, a cell does not need to move before judging the direction of a gradient. Zigmond proposed that amoeboid cells, unlike bacteria, utilize a spatial sensing mechanism to detect a chemical gradient and to guide their movement (Zigmond, 1974). Since then, the model system *D. discoideum* has been used to raise fundamental questions in eukaryotic chemotaxis, propose hypotheses of gradient sensing, discover essential components involved in chemotaxis, and develop techniques to study chemotaxis.

## A GPCR-Mediated Signaling Network for Eukaryotic Chemotaxis

The receptor for chemoattractant cAMP in *D. discoideum* was discovered as a GPCR in 1988 (Klein et al., 1988). In the ensuing years, receptors for fMLP, C5a, and IL-8 (CXCL8) in human leukocytes were also found to be members of the GPCR family (Boulay et al., 1990; Gerard and Gerard, 1991; Holmes et al., 1991; Murphy and Tiffany, 1991). These findings established the paradigm that chemoattractants of eukaryotic cells are detected by GPCRs. Many components involved in chemotaxis were discovered in *D. discoideum*, and their homologs were then found to play similar roles in the chemotaxis of leukocytes.

**FIGURE 1:**
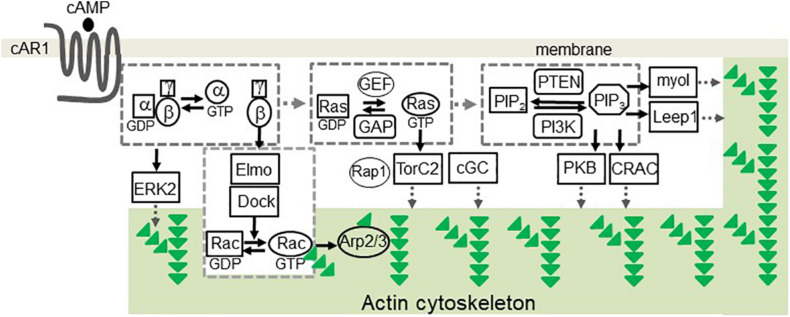
cAR1-mediated signaling pathways involved in chemotaxis in *Dictyostelium discoideum*. The binding of cAMP to cAR1 triggered signaling events leading to the reorganization of the actin cytoskeleton. The events include the dissociation of heterotrimeric G protein into Gα and Gβγ subunits, the activation of Ras, PIP3 production. PIP3 recruits signaling components, including PKB, CRAC, myoI, and Leep1, to transduce signals. Activation of heterotrimeric G-proteins activates Erk2 signaling to mediate chemotaxis and phagocytosis. Free Gβγs associate with the Elmo/Dock complex to activate Rac. Ras-GTP activates Rap1, TorC2 complex, and cGC. These signaling events regulate the actin cytoskeleton for cell migration. Green triangles represent G-actin molecules.

Over the years, pathways involved in the cAMP receptor (cAR1)-mediated cell migration have been identified in *D. discoideum* (Figure 1). The binding of cAMP to cAR1 induces dissociation (activation) of heterotrimeric G proteins into Gα2 and Gβγ subunits (Firtel et al., 1989; Janetopoulos et al., 2001; Ueda et al., 2001), which, in turn, activate downstream signaling components to control the reorganization of the actin cytoskeleton for chemotaxis (Gerisch et al., 1993). Free Gβγ subunits activate small G protein Ras proteins (Sasaki et al., 2004; Charest et al., 2010; Kortholt et al., 2011; van Haastert et al., 2017), which activate PI3K to convert PIP_2_ to PIP_3_ (Sasaki et al., 2004). Activation of cAR1 also induces a transient membrane dissociation of PTEN and then a reassociation of PTEN to the trailing back of the cell (Funamoto et al., 2002; Iijima and Devreotes, 2002). PTEN dephosphorylates PIP_3_ and converts it to PIP_2_, and local PIP_3_ levels on the membrane provide an intracellular cue to recruit proteins containing pleckstrin homology (PH) domains from the cytosol to the membrane (Funamoto et al., 2002; Iijima and Devreotes, 2002; Matsuoka and Ueda, 2018). The recruited proteins include cytosolic regulator of adenylyl cyclase (CRAC) (Insall et al., 1994; Parent et al., 1998), Akt/PKB (Meili et al., 1999), myosin I (Chen et al., 2012), and Leep1 (Yang et al., 2021), each of which plays a role in regulating the reorganization of the actin cytoskeleton. It is still not clear how CRAC and Akt/PKB binding to PIP_3_ regulate the actin cytoskeleton. PIP_3_ binding proteins of myosin I, an actin-based motor protein, and Leep1 promote actin polymerization at the leading front of a migrating cell. Three myosin-I proteins, myosin ID, IE, and IF, bind to PIP_3_ and therefore are recruited to the leading edge of a cell to promote actin-polymerization for chemotaxis (Chen et al., 2012). Recently, it was found that Leep1 interacts with PIP_3_ and the Scar/WAVE complex to control the actin cytoskeleton at the leading edge of a migrating cell (Yang et al., 2021). In addition, several PIP_3_-independent pathways have been implicated in the chemotaxis of *D. discoideum*. One pathway is that the activation of G proteins mediates cGMP signaling that controls myosin phosphorylation and chemotaxis (Bosgraaf and Van Haastert, 2002; Kortholt et al., 2011). The second one is a GPCR-mediated mTORC2 and AKT signaling (Charest et al., 2010). Upon the activation of cAR1, the small G-protein Ras is activated and becomes Ras-GTP, and the small G-protein RacE is phosphorylated by GSK3. Ras-GTP, RacE-P, and mTORC2 form a signaling complex to promote AKT phosphorylation, which regulates cell migration (Senoo et al., 2019). In addition, activation of the small G-proteins RasC/G and Rap1 transduces signals to mTOR2 for chemotaxis (Khanna et al., 2016; Tariqul Islam et al., 2019). The fourth pathway is that the free Gβγ subunit associates with an ELMO/Dock complex to activate Rac leading to actin polymerization for cell migration (Yan et al., 2012). Finally, activation of cAR1 induces the phosphorylation of Erk2 that mediates signaling for both chemotaxis and phagocytosis (Hadwiger and Nguyen, 2011; Schwebs et al., 2018; Nichols et al., 2019; Tariqul Islam et al., 2019).

Chemoattractant GPCR-mediated signaling mechanisms for the regulation of chemotaxis have also been studied in leukocytes. Many molecular mechanisms involved in GPCR-mediated chemotaxis are evolutionarily conserved in *D. discoideum* and mammalian cells. Activation of GPCRs by chemoattractants triggers dissociation (activation) of heterotrimeric Gi proteins into Gαi and Gβγ subunits (Neptune and Bourne, 1997), which control several signaling pathways. Activation of Gi-proteins produces free Gβγ that activates PI3Ks to generate PIP_3_, regulates mTORC2, and produces active forms of small G-proteins (Servant et al., 2000; Suire et al., 2006; Liu et al., 2010), including Ras and Rac, to regulate the reorganization of the actin cytoskeleton. Leukocytes express several PIP_3_ phosphatases, such as PTEN and SHIP, that are involved in the regulation of chemotaxis (Nishio et al., 2007; Heit et al., 2008). In addition, a pathway consisting of CXCR4/Gi, ELMO1/Dock180, and Rac appears to play a role in CXCL12-mediated chemotaxis and metastasis of breast cancer cells (Li et al., 2013). Another GPCR-mediated pathway of PLC, PKC, and PKD is involved in the regulation of chemotaxis of neutrophils (Xu et al., 2015).

Chemotaxing cells, including *D. discoideum* and neutrophils, are morphologically polarized with leading pseudopods and trailing ends (Devreotes and Zigmond, 1988). Many components involved in chemotaxis are asymmetrically distributed in these polarized amoebae (Parent and Weiner, 2013). Eukaryotic chemotaxis is a complicated cell behavior that conceptually consists of three interconnected cellular processes: gradient sensing, cell polarization, and cell motility. Advances in fluorescence microscopy have allowed us to visualize subcellular localization of cAR1 and other signaling components in live *D. discoideum* cells since the 1990s. These studies shed new light on the question of how eukaryotic cells generate directional biochemical responses in response to a chemical gradient.

## Gradient Sensing

Over the years, researchers have proposed different models, which involve either a temporal-sensing or a spatial-sensing mechanism (Zigmond, 1974; Parent and Devreotes, 1999; Rappel et al., 2002), to explain how eukaryotic cells detect and respond to chemical gradients. Many concepts have been developed and tested in the studies of *D. discoideum* chemotaxis. For example, one early hypothesis was that the accumulation of chemokine receptors at the front of a cell is essential for chemotaxis (Nieto et al., 1997). In 1997, cAR1 receptors were shown to be uniformly distributed on the membrane of chemotaxing *D. discoideum* cells (Xiao et al., 1997), and later, chemokine receptors were found to be the same on the surface of migrating neutrophils (Servant et al., 2000). In the ensuing years, studies showed that the *D. discoideum* cells can detect cAMP gradients to generate polarized responses even when cAR1 and its G-proteins are uninformedly distributed on the membrane (Parent et al., 1998; Jin et al., 2000). With the development of live-cell fluorescence microscopy, the spatiotemporal dynamics of key signaling events could be visualized (Gerisch and Muller-Taubenberger, 2003; Miyanaga et al., 2018). One breakthrough was the discovery that the chemoattractant sensing machinery works in cells without polarity and mobility (Parent et al., 1998). Specifically, *D. discoideum cells* become non-polar and immobile upon treatment with latrunculin, an inhibitor of actin polymerization. In latrunculin-treated cells, cAR1 and G proteins are uniformly distributed around the cell surface and PHcrac-GFP, a fluorescent probe for PIP_3_, is uniformly distributed in the cytosol (Parent et al., 1998; Jin et al., 2000; Matsuoka et al., 2006). When suddenly exposed to a cAMP gradient, a cell responds by initially inducing a transient PIP_3_ increase followed by a decrease around the membrane (a temporal adaptation) and producing a highly polarized distribution of PIP_3_ (a spatial amplification) in less than 2 min (Xu et al., 2005). During the application process, the membrane-bound PTENs gradually translocate from the front to the back (Iijima and Devreotes, 2002; Xu et al., 2007). The cAR1/G protein-controlled PIP_3_ responses display the key features of a spatial mechanism of gradient sensing. To truly understand gradient sensing mechanisms in chemotaxis, it is necessary to identify all regulatory components and to connect molecular mechanisms in a signaling network with a complexity that reflects that of chemotactic cells.

Over the last 20 years, many models have been proposed to explain GPCR-mediated gradient sensing and chemotaxis of eukaryotic cells (Parent and Devreotes, 1999; Postma and Van Haastert, 2001; Rappel et al., 2002; Van Haastert and Devreotes, 2004; Levine et al., 2006; Meier-Schellersheim et al., 2006; Iglesias and Devreotes, 2012; Takeda et al., 2012; Nakajima et al., 2014; Cheng and Othmer, 2016; Li et al., 2020). Most models were constructed by writing mathematical equations “by hand,” which is defining each reaction for molecule complex formation, association/de-association, or enzymatic transformation separately, and the tasks are time-consuming and very difficult due to a large number of reaction equations. It is almost impossible for experimental biologists to complete the tasks by themselves. The computer software packages have been developed and improved to allow researchers to construct signaling networks and to run simulations without dealing with mathematical equations. We hope that the software will be widely used by biologists to develop and analyze computational models that parallel live-cell experiments without writing reaction equations themselves, so more researchers can construct, examine, and validate their models. Future studies are needed to identify missing links and to understand how these components interact in a signaling network in time and space in a cell to achieve gradient sensing and chemotaxis.

## Signal Relay and Self-Generated Gradients

How eukaryotic cells migrate over long distances toward the source of chemoattractants is an interesting question. Eukaryotic cells guide their movement by spatially detecting chemical gradients. Because diffusible gradients diminish quickly over a distance, eukaryotic cells have developed systems to generate signals and move collectively as a group of cells. This process has been studied in *D. discoideum*. *D. discoideum* amoebae ingest bacteria as food in the soil. When food is depleted, *D. discoideum* cells stop dividing and enter a developmental process during which individual cells aggregate and form multi-cellular structures (Devreotes, 1994). Within a few hours following starvation, a few cells spontaneously secrete cAMP. cAMP acts as a chemoattractant binding to cAR1 that leads to the dissociation of the heterotrimeric G-protein into Gα2 and Gβγ and the activation of the adenylyl cyclase A(ACA) that converts ATP into cAMP, most of which is secreted to relay chemotactic signals to neighboring cells. These cells respond to cAMP stimuli by moving toward the center (chemotaxis) and secreting additional cAMP (signal relay). The net result is that cAMP waves spread outward through a population of 10^6^ cells as the cells moving toward the center. The cells migrate collectively toward secreted cAMPs in a head-to-tail fashion (streaming) and form an aggregation territory of about 0.5–1 cm in radius. It was found that ACA is not required for chemotaxis of individual cells, but ACA, specifically its asymmetric localization, is essential for cells to align head-to-tail in a stream and to migrate together as a group into aggregates (Kriebel et al., 2003).

A molecular system to relay chemotactic signals was discovered in neutrophils. Neutrophils are recruited to the site of an infection by primary chemoattractants, such as fMLP and C5a (Kolaczkowska and Kubes, 2013). After reaching the site, neutrophils respond to primary chemoattractants to produce and secrete secondary chemoattractants and recruit additional leukocytes to fight infection. A study showed that in response to an fMLP gradient, some neutrophils become polarized and migrate directionally, and they also release the secondary chemoattractant LTB4 (Afonso et al., 2012). All neutrophils, including those that have not yet responded to fMLP, respond to LTB4 and migrate toward the source of fMLP, indicating that LTB4 acts as a signal-relay molecule for neutrophils migrating collectively toward infection sites (Afonso et al., 2012; Lammermann et al., 2013). Interestingly, a recent study reported that *D. discoideum* cells can create chemoattractant gradients by degrading cAMP molecules and can achieve long-range chemotaxis by following these self-generated gradients (Tweedy et al., 2020). Mammalian cells may have systems to degrade attractants and make gradients for long-range migration toward the target sites.

## Signaling Events in Phagocytosis

Once they reach the infection sites, leukocytes catch and eliminate pathogenic bacteria via phagocytosis. Phagocytosis consists of several phases. (1) *Detection of the particles to be ingested*. Phagocytic receptors recognize specific molecules on the surface of bacteria. (2) *Activation of internalization*. Ligands binding to the receptors induce the reorganization of the actin cytoskeleton, which mediates the formation of a phagocytic cup to engulf the bacteria. (3) *Formation of the phagosome, a specialized vacuole*. The membrane protrusions of a phagocytic cup cover the particle and fuse at the distal ends creating a new vacuole containing the engulfed particle that then separates from the plasma membrane. (4) *Maturation of the phagosome into a phagolysosome*. The new phagosomes fuse with early endosomes and late endosomes that have accumulated V-ATPase and lysosomal-associated proteins on the membrane and luminal proteases; and then fuse with lysosomes to become phagolysosomes, where the ingested bacteria are degraded (Amulic et al., 2012; Flannagan et al., 2012; Buckley et al., 2019). This review will focus mostly on phases 1 and 2, which share similarities with chemotaxis, and will not cover phases 3 and 4 and instead recommend two excellent reviews (Boulais et al., 2010; Dunn et al., 2017).

Phagocytosis begins when a particle interacts with phagocytic receptors. The activation of these receptors initiates intracellular signaling pathways that lead to the reorganization of the actin cytoskeleton, the extension of pseudopods, and the engulfment of the particles. In leukocytes, phagocytic receptor-mediated signaling events include activation of tyrosine kinases and PI3K that regulate the actin cytoskeleton (Greenberg, 1995; Underhill and Ozinsky, 2002; Uribe-Querol and Rosales, 2020). For Fcγ receptors, the initial intracellular signaling event is phosphorylation of tyrosine residuals of the receptors and association of the receptors containing immunoreceptor tyrosine-based activating motifs (ITAMs) with members of the Src family. The phosphorylated receptors and ITAMs serve as docking sites for tyrosine kinases, such as Syk, to activate downstream components leading to the reorganization of actin cytoskeleton for the engulfment of the particles. Activation of the Fcγ receptor also promotes PI3K activities that contribute to the engulfment. As mentioned earlier, PI3Ks may regulate actin cytoskeleton for pseudopod extensions for both cell migration and particle ingestion. For a long time, it was generally thought that phagocytes use GPCRs to detect soluble chemoattractants derived from infection sites and to migrate toward bacteria, and then switch to phagocytic receptors to bind the bacteria for phagocytosis. The social amoebae *D. discoideum*, which detect folic acid released from bacteria and pursue them via chemotaxis and consume them through phagocytosis, have a simpler solution.

*Dictyostelium discoideum* cells do not encode orthologs of any known phagocytic receptors (non-opsonic- or opsonic-receptors) or tyrosine kinases (Goldberg et al., 2006), yet they are highly evolved as professional phagocytes (Dunn et al., 2017; King and Kay, 2019). In the 1990s, one study showed that a heterotrimeric G protein β subunit is required for both chemotaxis and phagocytosis (Peracino et al., 1998). Two other studies reported that either diffusible cAMPs or one yeast particle induced the accumulation of coronin, an actin-associated protein, in a leading pseudopod for either cell migration or the formation of a phagocytic cup to engulf the yeast (Gerisch et al., 1995; Maniak et al., 1995). These studies suggested heterotrimeric G-proteins are involved in signal transduction for the reorganization of actin cytoskeleton for both chemotaxis and phagocytosis. More than two decades later, it was discovered that *D. discoideum* cells utilize a GPCR (fAR1) linking to heterotrimeric G proteins to detect both diffusible folic acids released from bacteria and lipopolysaccharide (LPS) on the surface of the bacteria (Pan et al., 2016, 2018). Thus, this simple organism uses one GPCR/G protein machinery to regulate a signaling network for both “chemoattractant-mediated cell migration” to chase bacteria and “microbe-associated molecular pattern (MAMP)-mediated engulfment” to ingest bacteria. Interestingly, recent studies have suggested that neutrophils can utilize the chemoattractant GPCR/Gi signaling to mediate (Dunn et al., 2017) not only cell migration but also particle ingestion to fight against invading bacteria, indicating that mammalian phagocytes retain this mechanism to chase and interact with bacteria through the evolution (Wood Jr., Smith and Watson, 1946; Osei-Owusu et al., 2019; Wen et al., 2019).

## A Class C GPCR Is a New Members of Pattern Recognition Receptors

**FIGURE 2:**
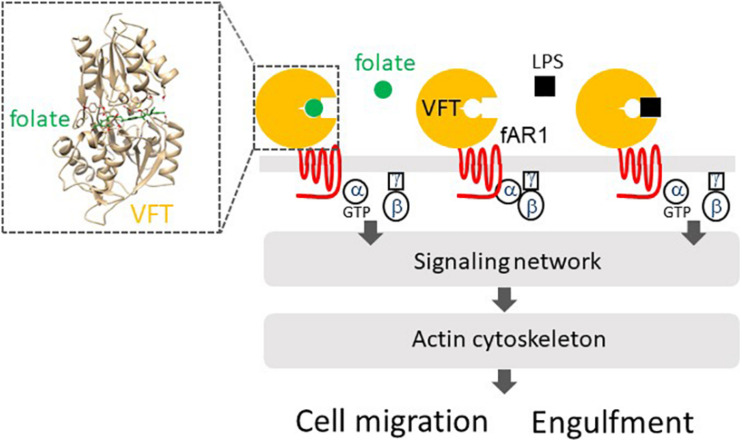
fAR1 binds to folate or LPS to activate heterotrimeric G-proteins that mediate a signaling network, which regulates the actin cytoskeleton for cell migration or particle engulfment. fAR1 contains a Venus-flytrap domain (VFT) for ligand binding. On the right box, Structural modeling and computational docking predict the extracellular domain of fAR1 folding into a VFT structure as the binding site for folate (green).

*Dictyostelium discoideum* amoebae utilize fAR1 and cognate G-proteins to detect folate for chasing bacteria and the immobile LPS on the bacterial surface for engulfing them (Figure 2). fAR1 contains a Venus-Flytrap (VFT) extracellular domain and belongs to the class C GPCR family (Pan et al., 2018). More than 800 human GPCRs have been identified so far and are classified into five main families: class A, which includes chemokine receptors, class B1, class B2, class C, and class F (Fredriksson et al., 2003; Foord et al., 2005). Based on phylogenetic sequence analysis and structure similarity, cAMP receptors (cAR1-cAR4) in *D. discoideum* constitute their own class (Saxe III, Johnson et al., 1991; Ginsburg et al., 1995; Moran et al., 2016), while fAR1 is a member of the class C family.

Class C GPCRs have a common structure consisting of an N-terminal extracellular Venus-flytrap (VFT) domain for ligand recognition and binding (Cao et al., 2009). VFT architecture is a typical structure of bacterial periplasmic binding proteins (PBPs) that sense small molecules and transport them into the cytoplasm (Borrok et al., 2009). A PBP consists of two globular domains connected by a small hinge region and exists in an open form in the absence of a ligand. The binding of a ligand induces a dramatic conformational change from an open to a close form, in which the ligand is clamped between the two lobes just like the carnivorous plant Venus-flytrap closing itself when a bug moving into the trap (Borrok et al., 2009). The class C GPCRs in humans are mainly composed of metabotropic glutamate receptors (mGluRs), gamma aminobutyric acid type B receptors (GABA_*B*_Rs), Ca^2+^-sensing receptors (CaSRs), taste receptors (T1Rs), pheromone receptors, and olfactory receptors (Cao et al., 2009). *D. discoideum* genome encodes 14 class C GPCRs, including fAR1 (Prabhu and Eichinger, 2006; Fey et al., 2013). The VFT domains of class C GPCRs in *D. discoideum* are closers to bacterial PBPs than those of human class C GPCRs (Cao et al., 2009). Interestingly, a bacterial PBP binds multiple ligands, while most human class C GPCRs expressed in the central nerve system bind only one natural ligand (Cao et al., 2009). However, the VFT in fAR1 retains the ability to recognize different molecules: folate released by bacteria and the sugar part of LPS (Figure 2), which is a major microbe-associated-molecular-pattern (MAMP) on the gram-negative bacteria (Pan et al., 2018). It appears is that the fusion of a PBP and an ancestral rhodopsin-like receptor (a class A GPCR) formed the common ancestor of the class C GPCRs, and fAR1 represents a prototype of class C GPCRs and is a new member of PRRs. Future studies will reveal the receptors of *D. discoideum* that recognize gram-positive bacteria and commune them as food.

## Human Leukocytes Acquired Features for Fighting Foreign Pathogens

**FIGURE 3:**
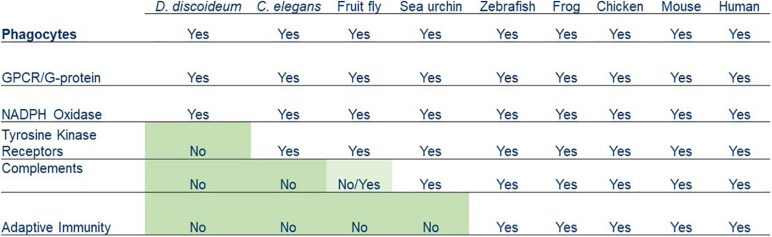
Summary of various phagocytosis components that have been acquired by phagocytes from simple organisms to humans.

Chemotaxis and phagocytosis can be observed in amoeboid cells from many eukaryotic organisms, including *D. discoideum*, *Caenorhabditis elegans*, fruit fly, sea urchin, zebrafish, frog, chicken, mouse, and human. Some molecular components involved in these two processes are found in the early life forms, such as protozoans that go back about 2.5 billion years. Other components are unique to higher vertebrates but bear similarities to those of invertebrates (Figure 3). Collectively, these links represent compelling evidence that human leukocytes evolved from ancient life forms and acquired new features over a billion years.

The major components involved in GPCR-mediated chemotaxis are evolutionarily conserved from *D. discoideum* to human cells. Earlier we have briefly mentioned the molecular components involved in a GPCR-mediated signaling network of eukaryotic chemotaxis. We know that the molecular components of the actin cytoskeleton and their regulators are evolutionarily conserved from amoebae of simple organisms to human leukocytes. We will not review these components and regulators of the actin cytoskeleton and instead recommend several outstanding reviews (Pollard and Borisy, 2003; Insall and Machesky, 2009; Devreotes and Horwitz, 2015).

*Dictyostelium discoideum* phagocytosis is not very different from humans (Dunn et al., 2017; King and Kay, 2019). After binding to a particle, amoebae of *D. discoideum* perform the internalization, formation of the phagosome, and maturation of the phagosome into phagolysosome. The *D. discoideum* genome encodes evolutionarily conserved components required for these processes, including particle internalization, phagosome formation, and maturation. For example, the *D. discoideum* genome encodes catalytic NOX (NADPH oxidase) subunits, which are homologs of NOX2 in humans. NOX2 generates superoxide by transferring an electron from cytosolic NADPH to O_2_ in the lumen of the phagosome. Reactions convert superoxide into reactive oxygen species (ROS), which contribute to the killing of internalized bacteria. *D. discoideum* and mammalian phagocytes use conserved strategies to kill bacteria. They both have V-ATPase for phagosome acidification. In addition, their phagosome acquires a series of proteases, hydrolases, and lysozymes to break down bacterial components. Interestingly, *D. discoideum* has a primitive innate immune system. In the multicellular slug stage, specialized phagocytic cells, sentinel (S) cells, circulate within the slug to trap, engulf, and kill bacteria. Recent studies revealed that S cells use Toll/interleukin-1 receptor (TIR) domain protein, TirA, to recognize bacteria, produce extracellular traps (ETs) and reactive oxygen species to kill bacteria in the slug of *D. discoideum* (Chen et al., 2007; Zhang et al., 2016).

Several features found in leukocytes for pathogen recognition are missing from the primitive phagocytes of *D. discoideum*. The *D. discoideum* genome does not encode complements, IgGs, or phagocytic receptors, such as tyrosine kinases/tyrosine kinase receptors (Goldberg et al., 2006; Cosson and Soldati, 2008). *D. discoideum* amoebae utilize a GPCR/G-protein machinery and signaling pathways for recognizing both the chemoattractant folic acid and a phagocytic cue LPS for chasing and ingesting *Klebsiella aerogenes* (Pan et al., 2016, 2018). Recent reports suggested that *D. discoideum* encodes lectins and the scavenger receptor, LmpB (Dinh et al., 2018; Sattler et al., 2018), but it is not clear how (or whether) they form a functional signaling pathway to mediate phagocytosis. *Caenorhabditis elegans* does not encode complements or adaptive immune system but expresses tyrosine kinases that have the potential to associate with scavenger receptors for phagocytosis (Stuart and Ezekowitz, 2008). Animals ranging from sea urchins to humans have a version of the complement system, and vertebrates from fish to humans have both a complement system and adaptive immunity. Since phagocytes from all organisms have chemoattractant GPCR/G-protein machinery, we propose that the GPCR-mediated signaling network is an ancient system for regulating the actin cytoskeleton for both chemotaxis and the engulfment of particles during phagocytosis. The molecular machinery required for phagosome formation and maturation also existed in ancient phagocytes. Although having the ability to distinguish of self from non-self, the simpler machinery of an ancient phagocyte might not work sufficiently when multicellular organisms encounter more complicated situations. New features were acquired by phagocytes of multicellular organisms to fight against various foreign microorganisms during the evolution. A receptor system that links to tyrosine kinases was added in *C. elegans*, a complement system was added in sea urchins and retained in animals with a fluid-filled body cavity or a circulatory system, and adaptive immunity was then acquired by vertebrates as the latest feature. Human phagocytes have evolved by retaining early features and acquiring new ones that have gradually increased the complexity of their machinery and enhanced their ability to fight foreign pathogens in different situations.

## Pathogens Hijack Receptors on Immune Cells

Throughout their long co-evolution with their eukaryotic hosts, infectious pathogens, such as bacteria and viruses, have developed strategies for evading and (or) invading the immune system (Alcami, 2003). For example, *Yersinia pestis*, the bacterial agent of plague, selectively kills immune cells, enabling its multiplication and systemic spread (Osei-Owusu et al., 2019). The plague receptor on human immune cells is FPR1, a chemoattractant GPCR of N-formyl peptides released from the bacteria. Once it has attracted immune cells, *Y. pestis* uses its cap protein in the type III secretion system (T3SS) to bind FPR1 and then delivers effector proteins to the host immune cells. The injected effector proteins inhibit host cell signaling and activate apoptosis of these host cells.

Many viruses have also exploited chemokine receptor systems for their entry and replication in the host cells (Alcami, 2003; Lisco et al., 2007). For example, the binding of the HIV envelope proteins to CXCR4 or CCR5 triggers membrane fusion and signal transduction and promotes the entry of HIV into T cells or macrophages (Berger et al., 1999). A previous study showed that HIV-mediated CXCR4/Gi signaling activates cofilin, which regulates actin dynamics and is known to be involved in cell migration, to facilitate remodeling of the actin cytoskeleton for viral entry (Yoder et al., 2008). Therefore, HIV hijacks chemokine receptor systems to actively promote its entry and its nuclear localization to infect and multiply in host cells. Large DNA viruses, such as herpesvirus, encode a set of proteins that mimic chemokines and chemokine receptors, or secreted proteins that bind chemokines. These viral proteins have various functions to modulate immune systems for viral spread and replication (Alcami, 2003).

Chemokines and their receptors are indispensable for time- and site-dependent trafficking of human phagocytes to fight pathogens in the human body. On the other hand, pathogens have exploited chemokines and their receptors by hijacking and reprogramming chemokine signaling systems for their entry and replication in human cells. One challenge in fighting infectious diseases is to develop therapies that efficiently stop replication and the spread of pathogens without compromising the effectiveness of the human immune system or hyper-activating the system.

## Concluding Remarks

In this review, we have highlighted some important findings in the research on chemotaxis and phagocytosis of *D. discoideum* and human phagocytes. We believe that the intricacies of human innate immune response can only be fully understood by analyzing simpler organisms. The discoveries made over the last 100 years on the social amoebae and human phagocytes have shed a light on our understanding of not only the basic evolution of the innate immune system but also problems of human disease. Future research on *D. discoideum* and new findings from this simple organism will further enrich our knowledge of innate immunity and human health.

## Author Contributions

All authors listed have made a substantial, direct and intellectual contribution to the work, and approved it for publication.

## Conflict of Interest

The authors declare that the research was conducted in the absence of any commercial or financial relationships that could be construed as a potential conflict of interest.

## Publisher’s Note

All claims expressed in this article are solely those of the authors and do not necessarily represent those of their affiliated organizations, or those of the publisher, the editors and the reviewers. Any product that may be evaluated in this article, or claim that may be made by its manufacturer, is not guaranteed or endorsed by the publisher.
